# Medical Education in London

**Published:** 1908-09-05

**Authors:** 


### MEDICAL EDUCATION IN LONDON.

The conditions of medical education in London, like the management of the hospitals themselves, have been evolved gradually and slowly by adaptation to the exigencies of various ages. Like the British Constitution, which is peculiar to itself in being altogether unwritten and uncodified, and the British Empire, which is alleged to have been acquired in successive fits of absence of mind, those essentially British institutions the London Medical Schools, are governed by no laws, except their own traditions, their financial necessities, and the general public opinion of the medical profession. They are theoretically private syndicates of interested persons associated together for gain; practically they are more often sources of loss than profit, and their working is to a very considerable degree only possible by a display of altruism which is not always appreciated to the full outside the ranks of the profession.

In olden days students of medicine were apprenticed for considerable periods to general practitioners and completed their education by a process of what was termed "walking the hospitals." That is, the budding practitioner attached himself to some teacher of eminence, commonly one of the staff of a hospital, for whom he paid directly certain fees for tuition. This teacher admitted him to the wards and there instructed him and his fellows in what we should now call a ward class. Moreover, a room or lecture hall somewhere in the vicinity of the hospital, but not in any way attached to it, served for the master to teach by lectures, anatomical demonstrations, and doubtless other methods, such knowledge as was then considered to form the proper curriculum. The reputation of a teacher was thus determined by a direct pecuniary standard; and rivalries between more and less successful colleagues on the hospital staffs were often bitter.

Such a system was of service only in the infancy of modern medicine and surgery. With ever-increasing specialisation, not merely in the different departments of the clinical science, but in anatomy, physiology, chemistry, physics, and the rest, it became impossible for one man to teach all subjects, and co-operation by the formation of definite medical schools became inevitable. But these schools were, as we know, kept separate from the hospitals themselves, although they grew up alongside, and although the two in time became so mutually dependent that neither could separate from the other without the total disorganisation of both. Still, in our typical British way, the changes brought about, if continuous, were gradual; there was no sharp landmark at which it became evident at once to all that a new system had definitely supplanted the old. So to-day the same nebulous haphazard plan still exists, though there are signs to be gathered that radical changes are possible in the future.

At the present time the student who intends to prosecute his studies in London must, before he can join a medical school, pass a preliminary examination in arts. He may also, if he pleases, study for and pass the various parts of his first professional examination at various public and other schools recognised as competent to offer such instruction, before he attaches himself to a strictly medical school; or he may even, and often does, surmount the second professional examinations at a university before coming to London for his clinical experience. The fees which he is charged for attending the lectures delivered in the school, and enjoying various other privileges including laboratory facilities, oral and revision ,classes, demonstrations, etc., are entirely a question to be settled by those who control the particular school, though of course the competition of several schools existing in one town brings about a fairly uniform tariff.

The increasing complexity of a medical education of late years has been associated with enormously increased cost owing to the requirements of laboratory equipment on a scale vastly greater than could ever have been foreseen. The result of this is that in many schools the receipts from students are not only insufficient properly to remunerate the teaching staff, but may be insufficient to remunerate them at all. The only remedy for such a state of things, namely, an increase of the fees, is out of the question, for two reasons: one is that they are already as high as the majority of students can afford, and the other that endowed colleges and universities in the North and Midlands, have already attracted many of those who formerly came to London, and that their charges are already much lower than in the metropolis. The London schools are thus between the Devil and the Deep Sea indeed. As we have already noted in these pages (The Hospital, July 25, p. 433), an attempt is to be made to subsidise them by collecting funds for their endowment by means of a central fund. Similar attempts have already been made on behalf of individual schools, with results only partially successful, and it remains to be seen what this latest attempt to solve their difficulties will lead to. For some reason or other a University seems to appeal more forcibly to extremely wealthy men as a subject for munificent endowment, than a mere medical school more or less loosely attached to a charitable institution such as a hospital. The fact is comprehensible enough when the exact position of the medical schools is borne in mind. They were in their inception and are still nominally private adventures, having no charter, no constitution as it were, no dignity of robes and degrees, Chancellors and Rectors, graduates and undergraduates, and all the rest of the proper appurtenances of a full blown University. Yet there are medical schools in London whose records in point of work done, services rendered to the community, and students instructed, can challenge comparison with those of many a University. "Men, not measures," has always been a cherished principle in our island commonwealth; and to men the London schools can certainly lay claim. Only their efforts have been greatly stultified of recent years by lack of funds, and unless this financial question can be satisfactorily settled somehow, it will become doubtful how long London will be able to maintain its proud pre-eminence of the past.

